# A case of giant main pulmonary artery aneurysm associated with complicated congenital heart disease and simultaneous chronic pulmonary artery dissection

**DOI:** 10.1186/s13019-020-01139-6

**Published:** 2020-05-12

**Authors:** Yue-bo Wang, Guang-wen Chen, Hong Pu, Hang Li

**Affiliations:** Department of Radiology, Sichuan Academy of Medical Sciences and Sichuan Provincial People’s Hospital, Qingyang District, Chengdu, 610072 Sichuan China

**Keywords:** Pulmonary artery aneurysm, Chronic dissection, Computed tomography, Angiography.

## Abstract

**Background:**

Pulmonary artery aneurysm (PAA), usually associated with congenital heart disease (CHD), is a very rare clinical condition. Pulmonary artery dissection (PAD) is considered the most life-threatening complication of PAA, and patients can progress from being asymptomatic to sudden death. We report the following case of PAA associated with complicated congenital heart disease and simultaneous chronic PAD. To our knowledge, few such complicated cases have ever been reported.

**Case presentation:**

A 36-year-old male presented to our hospital with a 10-year history of heart fatigue after activities and aggravated for 2 years. Computed tomography angiography (CTA) and echocardiogram showed a giant main pulmonary artery aneurysm (14 cm) with complicated congenital heart disease (a small patent ductus arteriosus, ventricular septal defects, aortic coarctation). Chronic PAD, which was mistaken for a pulmonary valve before operation, was detected during surgery.

**Conclusions:**

PAA associated with complicated CHD and simultaneous PAD is very rare, and its clinical symptoms are varied. Radiologists and surgeons should pay attention to determining whether this patient simultaneously had PAD when PAA was detected on preoperative CTA imaging.

## Background

Pulmonary artery aneurysm (PAA) is a rare clinical condition with an estimated incidence of 1 in 14,000 individuals [1]. The most serious risk of PAA is the formation of pulmonary artery dissection (PAD) or rupture, which can cause sudden death without symptoms of hemodynamic decompensation [2]. PAA complicated with chronic PAD is rare, and few studies have been reported [1]. PAA associated with chronic PAD and simultaneous complicated congenital heart disease (CHD) has never been reported in previous studies. We herein report a case of a giant main pulmonary artery aneurysm with chronic dissection and very complicated CHD.

## Case presentation

A 36-year-old male presented to us with PAA and CHD following echocardiographic detection in another hospital. He had a 10-year history of heart fatigue after activities and aggravated for 2 years without any examination or treatment. He exhibited no clinical symptoms other than heart exhaustion. He had no past medical or family history of any other related diseases.

**Fig. 1:**
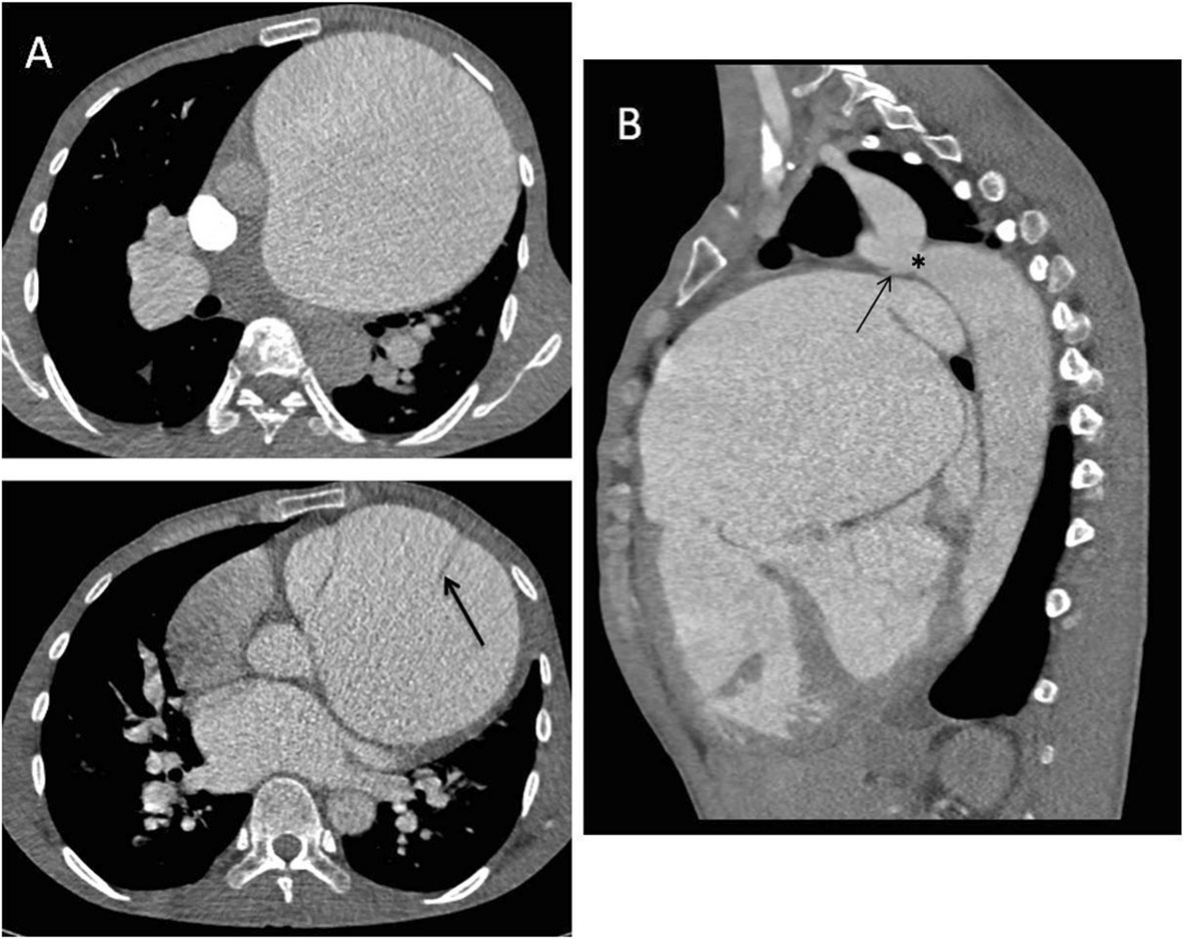
Computed tomography angiography. **a** Main pulmonary aneurysmal dilation with a diameter of 14 cm. **b** Aortic coarctation at the aortic isthmus and a small patent ductus arteriosus at the ductus arteriosus. **c** CTA showing pulmonary artery dissection

On admission, physical examination revealed left thoracic elevation. Rumbling pathological diastolic and systolic murmurs were heard along the sternal border in the third-fourth intercostal space in the subxiphoid and apical regions. Computed tomography angiography (CTA) showed a pronounced dilation of the pulmonary artery (PA) trunk, with a maximum diameter of 14 cm, and a diameter of 2.5 cm for the left and right PA (Fig. 1a). CTA also revealed ventricular septal defects (3 cm), a small patent ductus arteriosus, aortic coarctation (diameter 1.3 cm) (Fig. 1b), and distal arterial dilation. No intrapulmonary thrombi were observed. Echocardiogram showed mild mitral in the long axis of the left ventricle and tricuspid insufficiency in four-chamber view, severe pulmonary insufficiency and the dilated PA trunk in short axis view of great artery. Right heart catheterization demonstrated a systolic pulmonary artery pressure of 87 mmHg. Both preoperative CTA and echocardiogram failed to detect PAD.

**Fig. 2:**
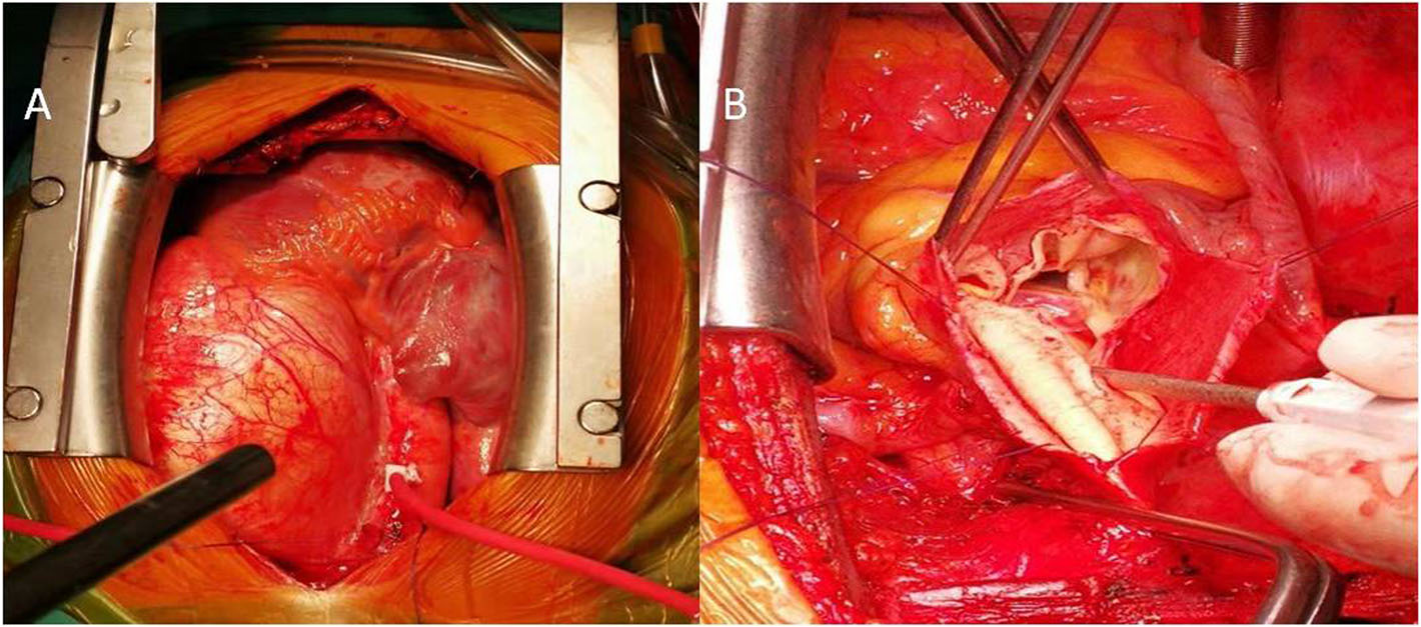
Intraoperative findings. **a** Intraoperative view of the main pulmonary aneurysm. **b** Intraoperative view of the main pulmonary dissection

The patient received combination sildenafil-bosentan therapy to reduce PA pressure conservatively 26 days prior to the operation. Subsequently, he underwent a very complicated surgery. A right atrial incision in the parallel atrioventricular groove and a pulmonary artery longitudinal incision were made, respectively. The atrial septal defect, tricuspid valve, ventricular septal defect, pulmonary valve were explored later. Chronic PAD was found intraoperatively, located 2.5 cm above the pulmonary valve with a length of approximately 10 cm (Fig. 1c, Fig. 2a-b). The ventricular septal defect was repaired with an autologous pericardium patch of 3.5 × 3.5 cm. Then, the mitral and tricuspid valvuloplasty were performed respectively with kay’s technique. Atrial septal incision and atrial septal defect were directly sutured with 3–0 prolene. Pulmonary valvuloplasty was performed with three pulmonary valve commissure suspension. The dilated pulmonary artery wall was partly cut off, and the PAD was folded with 5–0 prolene, then the pulmonary artery longitudinal incision was directly sutured with 5–0 prolene. Finally, the patient received artificial vascular graft bypass from the ascending aorta to descending aorta at the diaphragm plane. No residual shunt in the level of atrial septum and ventricular septum, and no significant regurgitation in the mitral and tricuspid valves were found by transesophageal echocardiography. Mild to moderate regurgitation was found in the pulmonary valve.

After the surgery, the patient was transferred to the Intensive Care Unit and subsequently to the general ward 7 days later. Postoperative echocardiogram showed a significant reduction in the size of PAA to 3.6 cm and PA pressure to 34 mmHg. His postoperative course was uncomplicated and without any special clinical complications. At 3 months after the operation, there was no significant increase in pulmonary artery diameter or in pulmonary artery pressure.

## Discussion

PAA is a rare condition, and its occurrence has been reported in only a few cases. It is defined as a focal dilatation of PA involving all 3 layers of the vessel wall with an upper limit of the main PA diameter of 29 mm and an upper limit of the interlobar PA of 17 mm [3]. The etiological factors of the formation of PAA include congenital causes, acquired causes, and idiopathic PAA. More than 50% of all cases have been associated with congenital heart disease (CHD) [4]. In decreasing order, the most frequent congenital heart defects included persistent ductus arteriosus, ventricular septal defects, atrial septal defects, a hypoplastic aortic valve and a bicuspid aortic valve in decreasing order. In addition, pulmonary valve regurgitation is also considered an independent factor in the formation of PAA [3]. Pulmonary artery hypertension (PAH) has been implicated as an acquired cause of PAA [5]. As in the patient in this study, we deduced that the giant main PAA was caused by CHD according to etiological factors. There are no clear guidelines available on the treatment of PAA as its incidence is rare. Kreibich et al. suggested a diameter threshold for surgery of the main PAA of > 5.5 cm with recommended indications, including the compression of adjacent structures, thrombus formation, an increase in the diameter of the aneurysm (≥5-mm) over 6 months, the appearance of clinical symptoms, the evidence of valvular pathologies or shunt flow, and the verification of PAH, which are life-threatening complications [4].

PAD occurs as a result of the formation of a tear through the intima into the mid- or deep media. The etiological factors of PAD are the same as those of PAA, and PAH is identified as the main disease leading to PAD [6]. PAD is considered a life-threatening complication of PAA and should be taken into management. Considering the lethality of PAD, it should be carefully diagnosed and monitored. Fernando et al. reviewed whether patients with PAD were diagnosed antemortem, and 70.5% of diagnosed patients survived [7]. Therefore, immediate surgery is recommended once PAD is detected. In our case, preoperative CT and echocardiogram failed to detect PAD. Early diagnosis of PAD is very important as it progresses to become chronic, which is often mistaken for a pulmonary valve. Therefore, if PAA is detected in a patient on preoperative CTA, radiologists and surgeons should pay attention to determine whether this patient simultaneously has PAD, regardless of whether the patient has severe clinical symptoms. Multiplanar reconstruction imaging can help radiologists differentiate PAD from pulmonary valves [8].

Together with our patients, only 14 cases of chronic PA dissection have been reported in patients with PH [9]. There are some reports regarding PAA without PAD and CHD [1, 10], PAA with CHD [11], and PAA with acute and chronic PAD [1, 12]. However, to the best of our knowledge, this is the first case of PAA associated with simultaneous chronic PAD and complicated CHD (a small PDA, ventricular septal defects, aortic coarctation) without severe clinical symptoms. Moreover, this is the second largest PAA (diameter of 14 cm) ever reported in the literature. A previous study by Fan et al. reported the largest PAA, with a diameter of 17 cm [13].

## Conclusions

PAA associated with simultaneous complicated CHD and PAD is very rare, and its clinical symptoms are varied. We have presented a unique case with serious complications. The report suggested that radiologists and surgeons should pay attention to determine whether this patient simultaneously had PAD when PAA was detected on preoperative CTA imaging.

## Data Availability

All the data and images are available for review by the Editor-in-Chief of this journal.

## Abbreviations

PAA: Pulmonary artery aneurysm
PAD: Pulmonary artery dissection
CHD: Congenital heart disease
CTA: Computed tomography angiography
PA: Pulmonary artery
PAH: Pulmonary artery hypertension
